# Modeling frequency-dependent action potential failures in CA3 pyramidal cell axons

**DOI:** 10.1186/1471-2202-13-S1-P115

**Published:** 2012-07-16

**Authors:** Ximing Li, William R Holmes

**Affiliations:** 1 Department of Biological Sciences, Neuroscience Program, Ohio University, Athens, OH 45701, USA

## 

When CA3 pyramidal cell axons are stimulated repetitively at low frequencies (10-20 Hz) the fiber volley detected in CA1 stratum radiatum is unchanged indicating faithful propagation. However at higher stimulation frequencies (50-100 Hz), the fiber volley is briefly potentiated with shorter latency, but quickly becomes depressed with longer latency. Antidromic action potentials in response to high frequency stimulation become reduced in amplitude, are wider and are prone to failure. To determine the mechanisms responsible for these experimental observations of frequency dependent action potential failure we turned to computational models.

We constructed a computational model of a CA3 pyramidal cell with detailed axon morphology taken from the http://Neuromorpho.org database [1]. Synaptic boutons were added to axon collaterals and diameters from the axon hillock to the initial segment to the early proximal axon were modified. Ion channels modeled include Nav1.2, Nav1.6, Kv and Km with kinetics and densities taken from Hu et al. (2009) [2]. The model included a mechanism to track extracellular potassium and intracellular sodium accumulation and the effect this has on the Nernst potentials. The size of the extracellular space for potassium accumulation was varied among simulations. Results were obtained for both low (20 Hz) and high (100 Hz) stimulation frequencies.

When stimulation frequency was 20 Hz, there were no propagation failures orthodromically or antidromically. When stimulation frequency was 100 Hz and the space for extracellular potassium accumulation was large, occasional action potential failures were observed in the antidromic direction as the action potential approached the diameter expansions beginning at the proximal axon. However no failures were observed in the orthodromic direction, even at diameter expansions at synaptic boutons. When the space for extracellular potassium accumulation was small, action potential failures became more frequent in the antidromic direction with action potentials first becoming wider and smaller and then dropping out for a period; this again occurred on the approach to the axon diameter expansion. In the orthodromic direction, action potential failures were observed at branch points. Interestingly, the action potential latency of successive action potentials in the train increased gradually until failure occurred. Sodium channel inactivation was found to increase gradually in cases where failure was eventually observed.

Our results suggest that ion accumulation, morphology and sodium inactivation kinetics interact to cause frequency dependent action potential failures in CA3 axons. At this time we cannot rule out additional contributions from synaptic transmission at boutons.

## References

[B1] NeuroMorpho.org database http://NeuroMorpho.org

[B2] Hu W Tian C Li T Yang M Hou H Shu Y Distinct contributions of Na(v)1.6 and Na(v)1.2 in action potential initiation and backpropagation Nat Neurosci 2009 12 8 996 1002 10.1038/nn.2359 19633666

